# Chemometric-guided chemical marker selection: A case study of the heat-clearing herb *Scrophularia ningpoensis*

**DOI:** 10.3389/fpls.2023.1153710

**Published:** 2023-03-17

**Authors:** Lung-Shuo Wang, Po-Jen Chen, Wen-Chi Cheng, Yu-Chia Chang, Mohamed El-Shazly, Lo-Yun Chen, Bo-Rong Peng, Chun-Han Su, Pei-Tzu Yen, Tsong-Long Hwang, Kuei-Hung Lai

**Affiliations:** ^1^ The School of Chinese Medicine for Post Baccalaureate, I-Shou University, Kaohsiung, Taiwan; ^2^ Cornucopia Traditional Medicine Clinic, Tainan, Taiwan; ^3^ Department of Chinese Medicine, Sin-Lau Hospital, Tainan, Taiwan; ^4^ Department of Medical Research, E-Da Hospital, I-Shou University, Kaohsiung, Taiwan; ^5^ Graduate Institute of Pharmacognosy, College of Pharmacy, Taipei Medical University, Taipei, Taiwan; ^6^ Research Center for Chinese Herbal Medicine, College of Human Ecology, Chang Gung University of Science and Technology, Taoyuan, Taiwan; ^7^ Graduate Institute of Health Industry Technology, College of Human Ecology, Chang Gung University of Science and Technology, Taoyuan, Taiwan; ^8^ Department of Pharmacognosy, Faculty of Pharmacy, Ain-Shams University, Cairo, Egypt; ^9^ Department of Food Science, College of Human Ecology, Fu Jen Catholic University, New Taipei City, Taiwan; ^10^ Jian Sheng Tang Chinese Medicine Clinic, Kaohsiung, Taiwan; ^11^ Graduate Institute of Natural Products, College of Medicine, Chang Gung University, Taoyuan, Taiwan; ^12^ Department of Chemical Engineering, Ming Chi University of Technology, New Taipei City, Taiwan; ^13^ Department of Anesthesiology, Chang Gung Memorial Hospital, Taoyuan, Taiwan; ^14^ PhD Program in Clinical Drug Development of Herbal Medicine, College of Pharmacy, Taipei Medical University, Taipei, Taiwan; ^15^ Traditional Herbal Medicine Research Center, Taipei Medical University Hospital, Taipei, Taiwan

**Keywords:** *Scrophularia ningpoensis* Hemsl., chemical markers, multi-informative molecular networking, ROS-dependent anti-inflammation, neutrophil

## Abstract

The selection of medicinal plants’ chemical markers focuses on bioactivity as the primary goal, followed by the nature of secondary metabolites, their stability, and availability. However, herbal medicines are valued for their complex and holistic pharmacological effects. A correct chemical marker can be carefully selected by a systematic clarification of their chemical-biological relationships. In the current study, the multi-informative molecular networking (MIMN) approach was employed to construct the anti-inflammatory metabolomic pattern of a heat-clearing herb, *Scrophularia ningpoensis* Hemsl. (*S. ningpoensis*). The MIMN molecular families characterized by cinnamic acid glycosides showed a higher bioactivity score compared with the other two major chemical classes (iridoid glycosides and iridoid-cinnamic acid glycosides). The Global Natural Product Social Molecular Networking (GNPS) and Reaxys database were used to assist in the putative annotation of eighteen metabolites from the bioactive and non-bioactive molecular families. The anti-inflammatory validation step was based on the detection of reactive oxygen species (ROS) generation by activated human neutrophils. All compounds from the bioactive MIMN molecular families dose-dependently inhibited the total ROS generation promoted by fMLF (IC_50_: 0.04–0.42 μM), while the compounds from non-bioactive MIMN clusters did not show any significant anti-inflammatory effect. The ROS-dependent anti-inflammatory activity of these cinnamic acid glycosides was attributed to their oxygen radical scavenging ability. The most abundant cinnamic acid glycoside, angoroside C (IC_50_: 0.34 μM) was suggested to be selected as a chemical marker for *S. ningpoensis*. In this study, the MIMN platform was applied to assist in the chemical marker selection of *S. ningpoensis*. The correct selection of markers will aid in the compilation and revision of herbal monographs and pharmacopeias resulting in the precise analysis and classification of medicinal plants on a scientific basis.

## 1 Introduction

Traditional Chinese Medicine (TCM) is a discipline that focuses on theories and practices of disease prevention, diagnosis, treatment, and rehabilitation (Lu et al., 2004). The prescription of unique herbal formulas containing a specific combination of ingredients is one of the valued features of TCM. These formulas are the products of TCM practitioners’ expertise through millennia. As the Chinese proverb goes, “formulating a prescription and using medicine is like dispatching troops”. Each medicine (herb) has its medicinal nature, cold or hot, and a specific range of actions. A precise and effective prescription can be only complied with a clear understanding of the characteristics of each herbal ingredient. Therefore, the thorough clarification of the chemical and pharmacological properties of TCM herbs using modern scientific approaches is currently the primary goal of TCM translational research.

The advancement of TCM evidence-based research can be achieved using modern spectroscopic techniques such as “MS/MS molecular networking (MN) approaches” (Watrous et al., 2012). MN is a visual computational strategy that can be intuitively implemented using LC-MS/MS data (Watrous et al., 2012). The visual computational strategy of MN is based on comparing the theoretical MS/MS spectra aiming to establish a relative network. Similar structures are clustered together according to their similar MS/MS spectra. This approach can be applied to explore the chemical diversity of herbal plants. The combination of MN with bioassay-guided chromatographic profiling can reveal the full picture of the chemical-bioactivity relationship by revealing which compounds are more likely to contribute to the bioactivity of the selected herbs. It also allows the identification of known or undiscovered compounds.

TCM suggests that a “heat syndrome” is a type of disease that occurs when the Yang Qi of the body becomes abnormally hyperactive (Dashtdar et al., 2016). In addition to common fever, there are many heat signs such as thirst, flushing, yellow urine, red tongue, yellow tongue fur, and quickened pulse (Zhu et al., 2017). To treat heat syndrome, herbs with cold and cool natures are used. There are many types of heat-related disorders, generally divided into exterior and interior disorders based on the representation and location of the “heat symptoms”. Herbs that can clear away interior heat are defined as heat-clearing herbs (HCHs). Recent scientific findings linked the clinical application of HCHs with the “anti-inflammatory” effect of modern pharmacology based on systematic literature reviews and network pharmacology analysis (Wang et al., 2018). One review found that the heat-clearing effect of *Scutellaria barbata* (a typical HCH) could be attributed to its anti-inflammatory and hepatoprotective activities, whereas its detoxifying effects might be due to its anti-microbial components of neo-clerodane diterpenoids and flavones (Wang et al., 2020). Although numerous scientific reports pointed out the anti-inflammatory pharmacological relevance of HCHs, their secondary metabolite content is still unknown. Therefore, we propose an MN strategy to explore the chemical diversity of these heat-clearing herbal medicines.

A typical HCH, *Scrophularia ningpoensis* Hemsl. (*S. ningpoensis*), was selected in the current study. *S. ningpoensis* has been used as a medication or tea by TCM practitioners (Lee et al., 2021). It is used in treating laryngitis, swelling, fever, constipation, and neuritis, as well as in boosting immunity (Shen et al., 2012; Meng et al., 2018). Regarding its heat-clearing ability, *S. ningpoensis* inhibited LPS-induced RAW 264.7 macrophages through mediating inducible nitric oxide synthase (iNOS), interleukins (IL)-1β and -6 (Byun et al., 2005). From the Pharmacopoeia of the People’s Republic of China (Pharmacopoeia Commission of the Ministry of Public Health, People’s Republic of China, 2020) and Taiwan Herbal Pharmacopeia (Ministry of Health and Welfare, Taiwan, 2021), two iridoid glycosides (harpagide and harpagoside) were selected as the chemical markers and were believed to be the main bioactive compounds related to the anti-inflammatory effect of *S. ningpoensis*. However, no reports investigated their effects on activated human neutrophils, which play a crucial role in various inflammatory diseases (Hakkim et al., 2011; Lai et al., 2021). Therefore, the *in vitro* anti-inflammatory (anti-reactive oxygen species (ROS) generation) assessment in activated neutrophils was used together with MN to construct a strategic workflow to reveal the anti-inflammatory chemical markers of this HCH.

## 2 Materials and methods

### 2.1 Chemicals and reagents

Cytochalasin B (CB), cytochrome *c*, dextran, dimethyl sulfoxide (DMSO), *N*-formyl methionyl-leucyl-phenylalanine (fMLF) were obtained from MilliporeSigma (St. Louis, MO, USA). Horseradish peroxidase (HRP) was purchased from Thermo Fisher Scientific (Waltham, MA, USA). Ficoll-paque plus was procured from GE Healthcare Systems (Little Chalfont, Buckinghamshire, UK).

### 2.2 Preparation of human neutrophils

Human donors (20–35 years old) provided blood by venipuncture. The protocol was approved and supervised by the Institutional Review Board (IRB) at Chang Gung Memorial Hospital (protocol code No. 202002493A3). The purification of neutrophils was achieved according to a previously reported method (Chiang et al., 2020). The protocol involved dextran sedimentation, hypotonic lysis, and Ficoll Hypaque gradient of erythrocytes. After the isolation of human neutrophils, they were suspended in a 50 mL centrifuge tube in calcium (Ca^2+^)-free and magnesium (Mg^2+^)-free HBSS buffer (KH_2_PO_4_: 60 mg/L; KCl: 400 mg/L; NaCl: 8,000 mg/L; NaHCO_3_: 350 mg/L; Dextrose: 1,000 mg/L). The pH was adjusted to 7.4 and the trypan blue exclusion method was used to examine neutrophils (> 98% viable cells). The assessment of neutrophils was achieved in HBSS (1 mM CaCl_2_ contained) at 37°C.

### 2.3 Sample extraction, preparation, and fractionation

The dried herbal materials of *Scrophularia ningpoensis* Hemsl. (*S. ningpoensis*) were provided by ChuangSongZong Pharmaceutical Co., Ltd (Kaohsiung City, Taiwan). To achieve the standardized extraction and to enhance extraction efficiency, an Accelerated Solvent Extractor (ASE 350) (Thermo Scientific, Waltham, MA, USA) was applied for the extraction of *S. ningpoensis* using 50% ethanol. The grounded *S. ningpoensis* powder (5 g) was prepared and added into a 66 mL stainless steel extraction cell, followed by automatic solvent filling, pressing, and heating. The extraction condition was set as 1500 psi (extraction pressure), 50°C (extraction temperature), 15 min (extraction time), 100% (solvent rinse volume), and 1 min (nitrogen purge time). The resulting products were concentrated under vacuum and lyophilized to afford *S. ningpoensis* 50% EtOH extract. For the preparation of analytical and biological samples, the extract was dissolved in methanol or dimethyl sulfoxide (DMSO) and was sterilized using a 0.45 μm micron filter.

For the primary fractionation, a Shimazu LC-2050 HPLC system (Shimazu, Kyoto, Japan) coupled with Galaksil EF-C18-H (5 μm, 120 Å, 10 × 250 mm, C18) column (Galak Chromatography, Jiangsu, China) was applied to fragment the *S. ningpoensis* extract. The eluting system was gradient made of a mixture of methanol (M) and water (W, containing 0.1% formic acid) starting from 5% M and ending with 100% M in 63 min (flow rate: 2 mL/min). The eluting solvent was individually collected to afford nine fractions (based on the retention time and chromatographic features). All collected fractions were subjected to further MS/MS fragmentation and bioassay.

### 2.4 Ultra-performance liquid chromatography-tandem mass spectrometry condition for non-targeted fragment ions collection

The MS^2^ data collection was carried out based on a Waters SYNAPT G2 LC/Q-TOF (Waters Corporation, Milford, MA, USA) system. The chromatographic separation before the MS spectra was performed using a C18 column of Waters Acquity UPLC BEH (Waters, 1.7 µm, 2.1 mm × 100 mm). The mobile phase was set as a MeCN (A, containing 0.1% formic acid)/water (W, containing 0.1% formic acid) gradient sequences: 0.01–7 min, 5–10% A; 7–15 min, 10–50% A; 15–17.5 min, 50–60% A; 17.5–25 min, 60% A; 25–27.5 min, 60–70% A; 27.5–28.5 min, 70–100% A; 28.5–30 min, 100% A. The flow rate was set up at 0.5 mL/min, and the temperature of the column part was maintained at 40°C in the oven. The extract (4 mg) was dissolved in 1 mL of methanol (5,000 ppm) and was filtered using a 0.45 μm membrane filter. The sample injection was applied automatically with an automatic syringe with a 5 μL volume per injection. The non-targeted MS^1^ and MS^2^ data were collected within the range of *m*/*z* 100–2000. The automated data-dependent acquisition (DDA) approach was applied in the MS^2^ scans, and the non-targeted selections of 5 precursor ions were fragmented with ramping of the collision energy from 10–50 eV. The acquired MS data were finalized by Waters MassFragment software (MassLynx4.1, Waters, MA, USA).

### 2.5 The global natural product social molecular networking-based molecular networking analysis

A GNPS web-based platform (https://gnps.ucsd.edu) was applied to analyze the output of the MS/MS molecular networking data (job ID: 7c306c4f928546dda206b658f7d38892, October 27^th^ of 2021). The MS/MS spectra were window-filtered according to the top five strongest ion peaks in the ± 50 Da window throughout the spectrum. A network was then created in which linkages between nodes were filtered by a cosine value above 0.70 and at least four matched peaks. The appeared nodes in the network were annotated based on the experimental MS^2^ fragmentations of the isolates. The molecular network was visualized and laid out using Cytoscape 3.8.2 (Cytoscape 3.8.2, NRNB, CA, USA).

### 2.6 Determination of superoxide anion (O_2_^•−^) generation

The superoxidase dismutase (SOD) inhibitable reduction of ferricytochrome *c* was used to determine O_2_^•−^ generation (Peng et al., 2021). Neutrophils (6 × 10^5^ cells/mL) were supplemented with ferricytochrome *c* (0.6 mg/mL), equilibrated at 37°C, and incubated for 5 min before treatment with the DMSO or the tested compounds. To magnify the reaction, 1 μg/mL CB was added and left (3 min) before activation (with 0.1 μM fMLF). Any change in the absorbance associated with the reduction of ferricytochrome *c* was monitored continuously at 550 nm (in 4.5 mL cuvette) using a spectrophotometer (U-3010, Hitachi, Tokyo, Japan).

### 2.7 Determination of ROS release

ROS were measured using luminol-enhanced chemiluminescence (Chiang et al., 2020). At 37°C, human neutrophils (7 × 10^5^ cells/mL) were mixed with luminol (37.5 μM) and horseradish peroxidase (HRP, 6 U/mL) for 5 min before treatment. DMSO or the tested compounds (0.001–10 μM) were loaded for 5 min before adding fMLF (0.1 μM) for activation. A 96-well chemiluminometer (Tecan, Infinite F200 Pro; Tecan Group, Männedorf, Switzerland) was applied to assess the chemiluminescence response.

### 2.8 Ferric reducing antioxidant power assay

The ferric-reducing antioxidant property was assessed through the reduction of ferric iron (Fe^3+^) to ferrous iron (Fe^2+^). The ferricytochrome *c* (0.6 mg/mL) was treated with the DMSO or the tested compounds. Any change in the absorbance associated with the reduction of ferricytochrome *c* was monitored continuously at 550 nm (in 4.5 mL cuvette) using a spectrophotometer (U-3010, Hitachi, Tokyo, Japan).

### 2.9 2,20-Azobis(2-methylpropionamidine) dihydrochloride ROS scavenging activity

MDGA or Trolox was preincubated with fluorescein (80 nM) in sodium phosphate (75 mM; pH 7.4). The AAPH (25 mM) was added to assess the ROS scavenging ability. The changes in the fluorescence absorbance were measured every 3 min for 120 min using a 96-well chemiluminometer (Tecan, Infinite F200 Pro; Tecan Group, Männedorf, Switzerland). The excitation and emission wavelengths were set as 485 nm and 535 nm, respectively.

### 2.10 Determination of lactate dehydrogenase release

The cell-free medium was used to assess the cytotoxicity against neutrophils as the ratio of the LDH released in total (Chen et al., 2019). At 37°C, the neutrophils (6 × 10^5^ cells/mL) were equilibrated and incubated for 5 min before treatment with the tested compounds (10 μg/mL). The treatment lasted for 15 min and the cells were then lysed using Triton X-100 (0.1%) at 25°C for 30 min. The LDH reagent was added and any changes in the absorbance at 492 nm (in 4.5 mL cuvette) were continuously monitored.

### 2.11 Bioactivity score calculation

The multi-informative molecular networking (MIMN) presented in the current study revealed the bioactivity, distribution, and putative chemical classes of specific precursor ion nodes, representing the bioactive probability of a group of compounds clustered in MIMN. To quantify this chemical structure-relevant bioactive probability, the bioactivity scores were calculated based on their bioactivity levels together with relative abundances (pie chart of the precursor nodes).

Bioactivity scores (BS) = anti-inflammatory level (1: inhibition rate ≥ 30%; 0: inhibition rate < 30%) × relative abundances (%)

### 2.12 Statistics

Statistical analysis was performed using Student’s *t*-test (GraphPad Software 9.0.2, San Diego, CA, USA) for all statistical calculations. *P* values < 0.05 were considered statistically significant.

## 3 Results and discussion

### 3.1 Multi-informative molecular networking platform for probing the chemical markers of heat-clearing herbs

**Figure 1:**
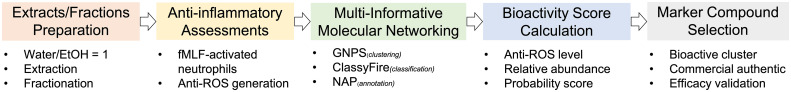
The multi-informative molecular networking (MIMN) platform for probing chemical markers of heat-clearing herbs (HCHs).

In the course of revealing the relationships between HCHs’ chemical constituents and their biological activities, we schemed a workflow based on MIMN and anti-inflammatory assessments in activated human neutrophils (**Figure 1**) (Chen et al., 2022). First, the extraction and fractionation were carried out using general chromatographic procedures. The MS^2^ data were collected and the anti-neutrophilic inflammation effect (inhibition of superoxide and ROS generation) of different fractions was determined. The obtained data were subjected to the Global Natural Product Social Molecular Networking (GNPS) analysis, which led to an overview of the bioactivity-coupled metabolomic spectral network. The estimation of the anti-inflammatory effect of each chemical cluster was executed through bioactivity score (BS) calculation based on the probability of compounds existing in the bioactive fractions. Based on BS, the bioactive and non-bioactive chemical clusters were suggested and the putatively annotated compounds from the bioactive clusters were purchased or isolated to afford the final experimental validation. The anti-inflammatory evaluation of the obtained compounds led to the selection of potential chemical markers.

### 3.2 Samples preparation and the development of a chromatographic anti-inflammatory profile of *S. ningpoensis*

Fractionation methods based on traditional column chromatography suffer from high levels of overlapping compounds, which reduces the accuracy of selecting bioassay-relevant biomarkers. Therefore, high-performance liquid chromatography (HPLC) was employed to ensure differentiation of the tested fractions during the bioassays. We performed the chromatographic fractionation of *S. ningpoensis* extract (prepared using ASE 350, 50% ethanol) based on the retention time differences and chromatographic features. The obtained nine fractions (SN1–9, at a concentration of 20 μg/mL) were assayed with activated neutrophils for their potential inhibitory activity of superoxide anion (O_2_^•−^) generation. The chromatographic anti-inflammatory profile was constructed (**Figure S1**). Among all fractions (at 20 μg/mL), the most potent fractions SN4 and SN7 significantly inhibited superoxide anion (O_2_^•−^) generation with 34.94% and 35.38% (*p* = 0.01), respectively (**Figure S1**, **Table S1**).

### 3.3 Characterizing *S. ningpoensis* constituents and their chemical classes using tandem mass spectroscopy and MIMN approach

Recent advances in spectroscopic techniques opened another page in the characterization of biologically active metabolites from nature (Jarmusch et al., 2021; Cheng et al., 2022; Su et al., 2022; Wang et al., 2022). To elucidate the relationships between the structural-dependent anti-inflammatory activities of *S. ningpoensis* fractions, the tandem mass MN strategy was incorporated with neutrophilic inhibitory properties (anti-O_2_^•−^) to characterize the potential anti-inflammatory constituents based on their MS/MS fragmentation features and chemical classes.

**Figure 2:**
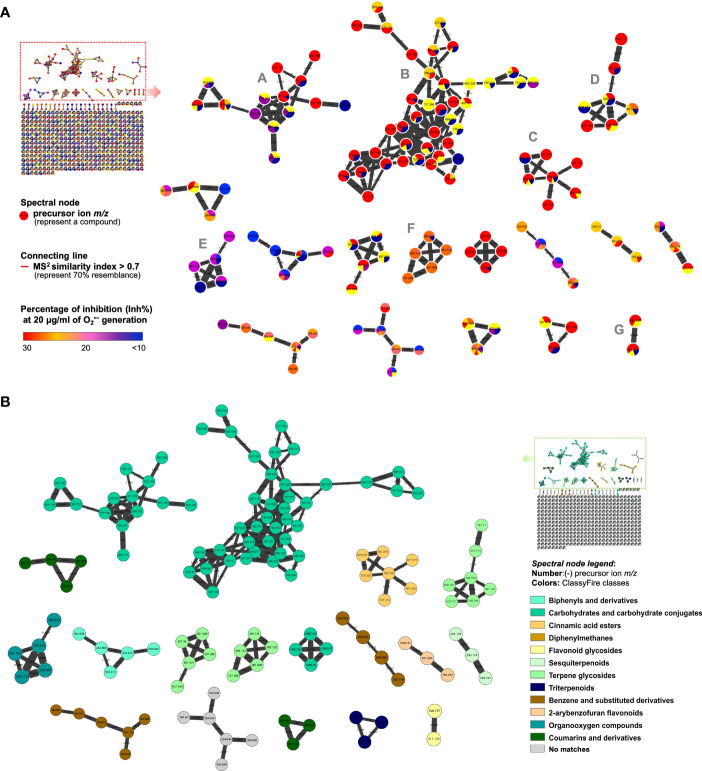
The MIMN illustrated the relationships between the metabolomic diversity and the anti-inflammatory properties of *Scrophularia ningpoensis* Hemsl. extract. **(A)** The established MIMN is based on the inhibition of O_2_^•−^ generation (20 μg/ml) in *N*-formyl methionyl-leucyl-phenylalanine (fMLF)-induced neutrophils and the spectral nodes were colored according to the levels of inhibition. The molecular clusters mentioned in the main text were marked with A-G. **(B)** The MIMN spectral nodes were labeled according to ClassyFire classes (categorized through their MS^2^ characteristics).

For the collection of MS^2^ fragmentation data, an ultra-performance liquid chromatography quadrupole time-of-flight mass spectrometry (UPLC-QTOF-MS) was employed using data-dependent analysis (DDA) mode. The acquired data and the similarity between fragments were further analyzed using the GNPS platform (https://gnps.ucsd.edu). The similar chemical skeletons were then clustered and visualized using Cytoscape software. In the established MIMN (**Figure 2A**) profile, the pie charts were tinted based on their O_2_^•−^ generation inhibitory levels (or distribution in each assayed fraction). The putative chemical classifications (**Figure 2B**) were further analyzed by ClassyFire software, indicating the majority of carbohydrate-conjugated metabolites in the *S. ningpoensis* extract. Moreover, the constituents from the *S. ningpoensis* extract were putatively annotated using Reaxys and GNPS database matches, which led to the identification of eighteen compounds (**Table 1**), belonging to carbohydrate conjugates, terpenoid glycosides, flavone glycosides, cinnamic acid esters, and organooxygen compounds.

**Table 1:** Identified metabolites from the extract of *Scrophularia ningpoensis* Hemsl.

No.	t*_R_* (min)	Proposed structure	Ion adduct	Precursor/ Product ion (*m/z*)	Molecular formula (Error in ppm)	Reference
1	0.92	*N*-Fructosyl Pyroglutamate	[M–H]^–^	290.089/161, 137, 135, 91	C_11_H_17_NO_8_ (5.2)	GNPS libraries
2	1.04	6-*O*-Feruloyl-*α*-D-fructofuranosyl-(2→1)-*α*-D-glucopyranoside	[M–H]^–^	517.155/337, 295, 265, 235, 193, 175	C_22_H_30_O_14_ (1.4)	(You-Hua et al., 2014)
3	1.11	Harpagide	[M–H]^–^	363.128/201, 183, 165, 113	C_15_H_24_O_10_ (3.0)	(Wang et al., 2017)
4	1.48	Acretoside	[M–H]^–^	487.144/382, 325, 265, 145	C_21_H_28_O_13_ (2.5)	(Tian et al., 2017)
5	1.81	Sibirioside A	[M+COOH–H]^–^	517.156/323, 189, 161, 147, 103	C_21_H_28_O_13_ (0.6)	(Jing et al., 2011)
6	3.00	Acteoside	[M–H]^–^	623.194/461, 161, 135, 113	C_29_H_36_O_15_ (5.8)	(Wu et al., 2006)
7	3.10	Isoacteoside	[M–H]^–^	623.194/461, 344, 161, 103	C_29_H_36_O_15_ (5.8)	(Xie et al., 2019)
8	3.58	Liquiritin	[M–H]^–^	417.122/265	C_21_H_22_O_9_ (8.2)	GNPS libraries
9	4.29	Scrophuside	[M–H]^–^	637.215/461, 193, 175, 113	C_30_H_38_O_15_ (2.7)	(Hua et al., 2014)
10	4.37	6′′-*O*-Feruloylharpagide	[M–H]^–^	539.177/337, 295, 265, 235, 193, 175, 134	C_25_H_32_O_13_ (0.9)	(You-Hua et al., 2014)
11	6.36	Poliumoside	[M–H]^–^	769.249/614, 593, 298, 193, 161	C_35_H_46_O_19_ (8.4)	GNPS libraries
12	6.61	Angoroside C	[M–H]^–^	783.266/607, 589, 193, 175, 160	C_36_H_48_O_19_ (6.4)	(Jing et al., 2011)
13	7.30	8-*O*-(*p*-Coumaroyl)harpagide	[M–H]^–^	509.163/163, 145, 119	C_24_H_30_O_12_ (5.7)	(Niu et al., 2009)
14	8.68	Isoangoroside C	[M–H]^–^	783.266/607, 589, 193, 175	C_36_H_48_O_19_ (6.4)	(Wu et al., 2010)
15	9.29	Scrospioside B	[M–H]^–^	679.225/499, 351, 291, 229, 165, 145	C_32_H_40_O_16_ (1.8)	(Zhang et al., 1992)
16	9.43	*β*-(3-Hydroxy-4-methoxyphenyl)ethyl-*O*-*α*-L-rhamnopyranosyl-(1→3)-*O*-[*α*-larabinopyranosyl-(1→6)]-(2-*O*-acetyl)-(4-*O*-caffeoyl)-*β*-D-glucopyranoside	[M–H]^–^	825.278/765, 649, 607, 589, 193, 175	C_38_H_50_O_20_ (4.5)	(You-Hua et al., 2014)
17	10.30	Cistanoside D	[M–H]^–^	651.229/475, 193, 175	C_31_H_40_O_15_ (0.2)	(Li et al., 2000)
18	12.84	Harpagoside	[M–H]^–^	493.177/345, 183, 165, 147	C_24_H_30_O_11_ (12.2)	GNPS libraries

### 3.4 Probing the anti-inflammatory constituents using MIMN-based bioactivity scores calculation

**Figure 3:**
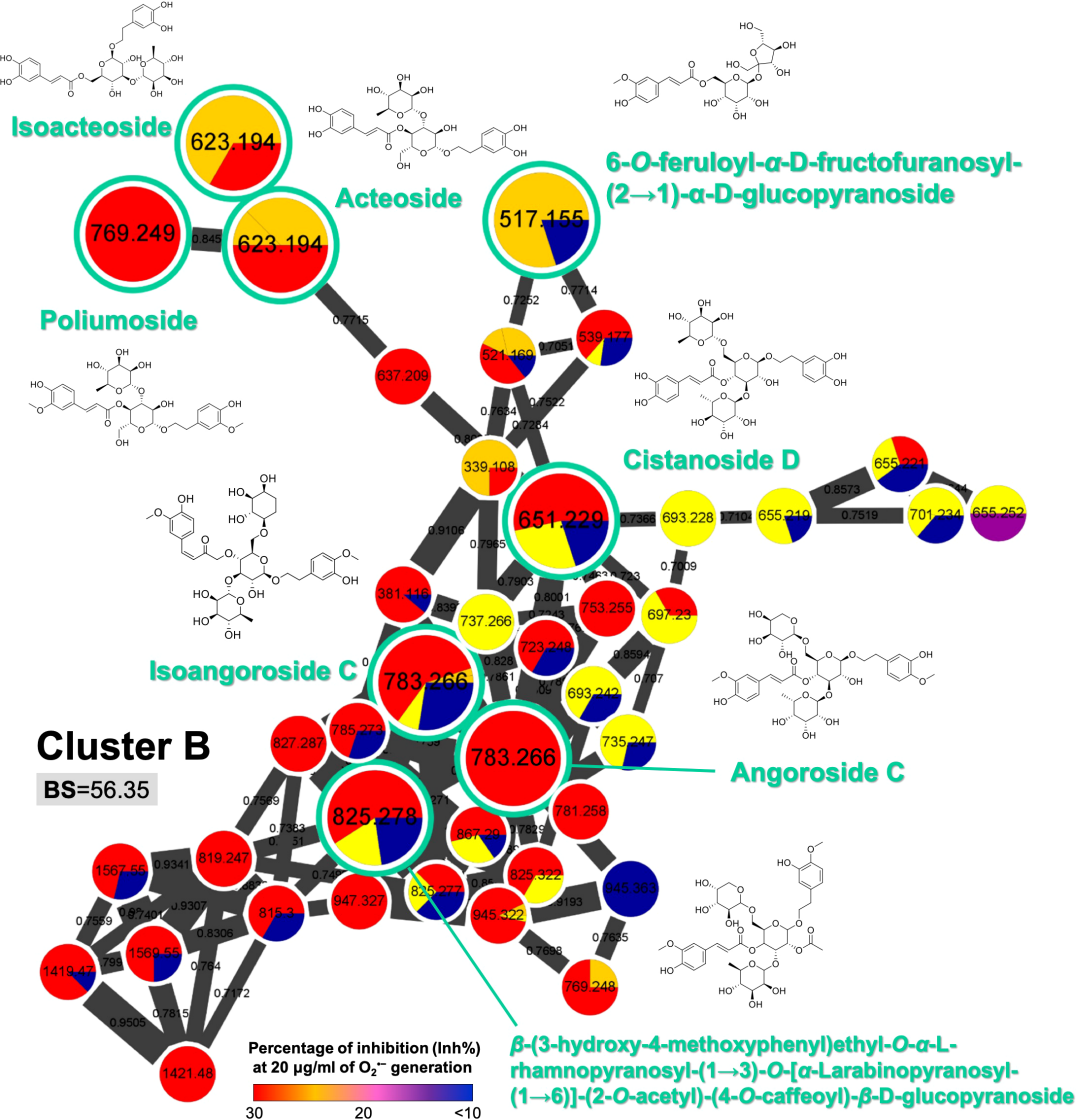
The selected MIMN cluster with a high bioactive score (BS ≥ 50) is a potential anti-inflammatory chemical group. The putative annotations were conducted based on the MS^2^ matching from GNPS and Reaxys databases.

To elucidate the relationships between the structural features and the anti-inflammatory properties, the bioactivity scores (BS) of each MIMN cluster (**Figure S2**) were calculated based on the anti-inflammatory level (1: inhibition rate ≥ 30%; 0: inhibition rate < 30%) and the relative abundances (%). Cluster B (**Figure 3**) showed the greatest chemical diversity as well as a high BS value (56.35). The metabolites that appeared in this cluster were characterized by multiple (two or three) carbohydrate units coupled with aglycone such as cinnamic acids.

**Figure 4:**
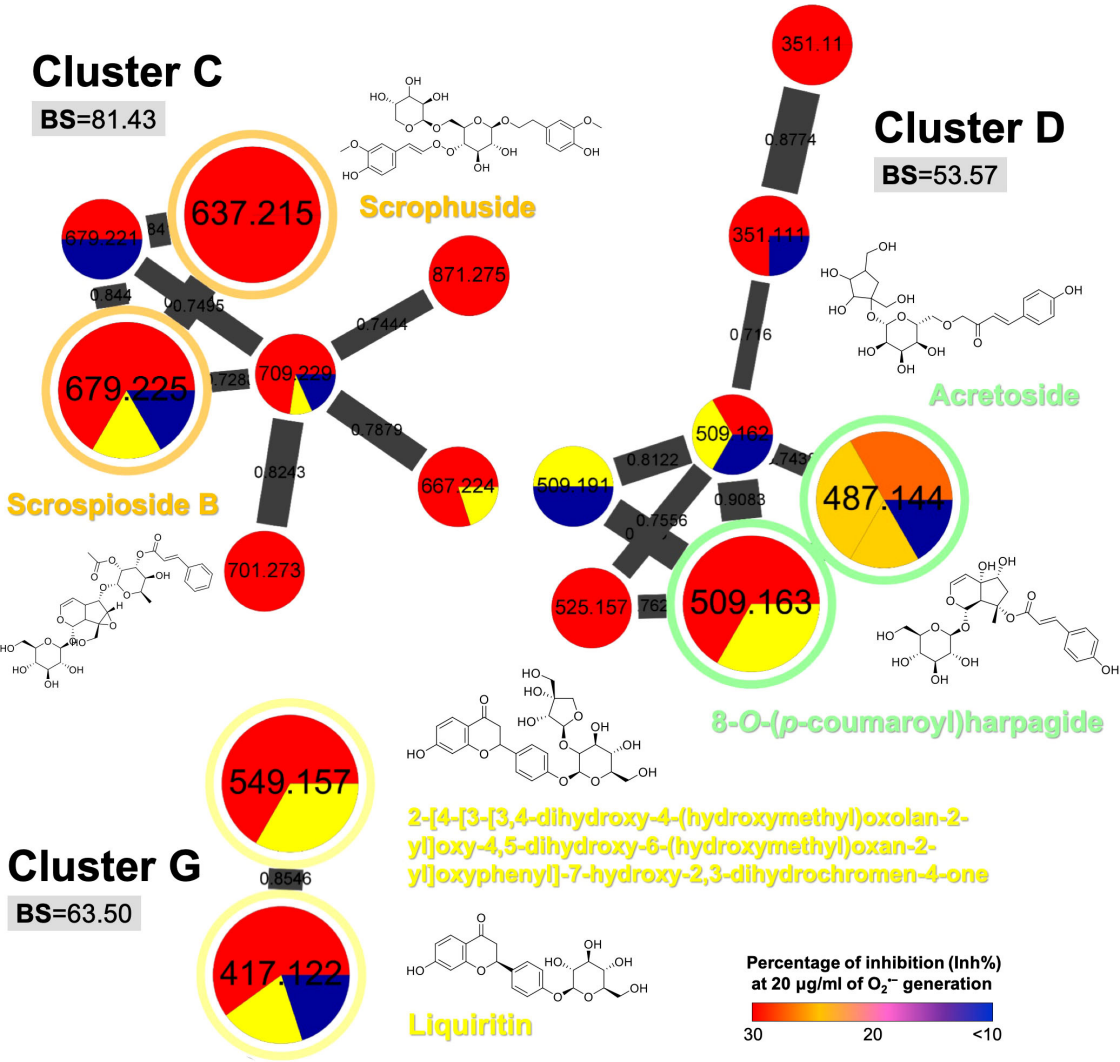
The selected MIMN clusters with high bioactive scores (BS ≥ 50) as potential anti-inflammatory chemical groups. The putative annotations were conducted based on the MS^2^ matching from GNPS and Reaxys databases.

Other three bioactive clusters with high BS (**Figure 4**) were found to be cinnamic acid eaters (cluster C: 81.43), terpene glycosides (cluster D: 53.37), and flavone glycosides (cluster G: 63.50). Although the chemical classification did not show distinctive identification among these bioactive groups, the cinnamic acid glycosides were still found to be the most potent metabolite class with anti-inflammatory effect (heat-clearance).

**Figure 5:**
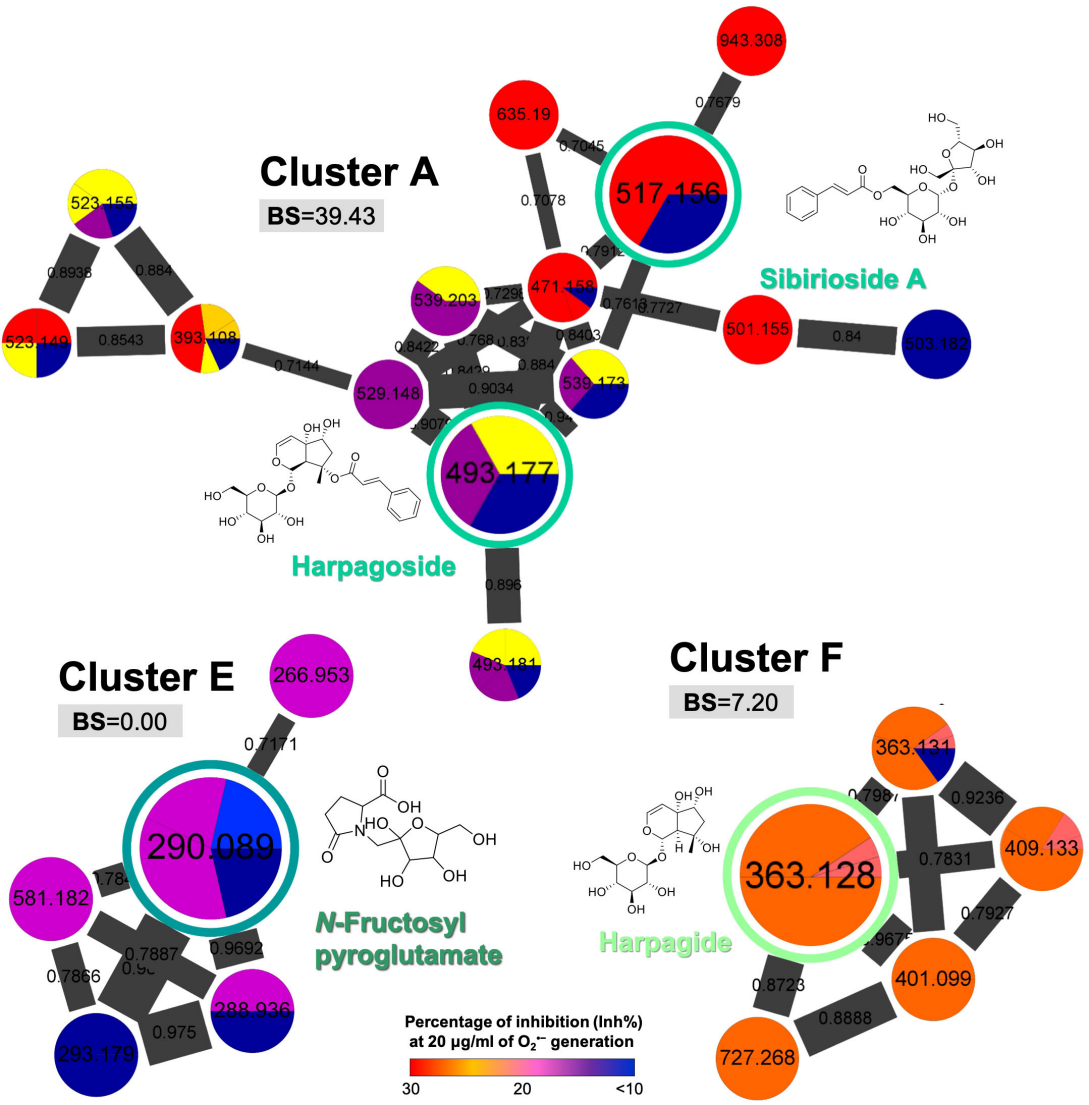
The selected MIMN clusters with low bioactive scores (BS < 50). The putative annotations were conducted based on the MS^2^ matching from GNPS and Reaxys databases.

On the other hand, the clusters such as iridoid-containing glycosides (cluster A: 39.43; cluster F: 7.20) and organooxygen compounds (cluster E: 0.00) showed relatively low BS (**Figure 5**), indicating that compounds other than cinnamic acid glycosides possessed less potential to be selected as a qualitative marker of *S. ningpoensis*. However, the two standardized chemical markers of *S. ningpoensis*, harpagide, and harpagoside, from the Pharmacopoeia of the People’s Republic of China (Pharmacopoeia Commission of the Ministry of Public Health, People’s Republic of China, 2020) and Taiwan Herbal Pharmacopeia (Ministry of Health and Welfare, Taiwan, 2021) are located in these non-bioactive clusters. Our obtained results should be further verified and validated by experimental bioassay assessments of the pure compounds.

### 3.5 MIMN-guided compounds selection and validation of anti-inflammatory markers

The MIMN profile and the follow-up annotations revealed that the three major constituents from *S. ningpoensis* extract were cinnamic acid glycosides (clustered in the groups of carbohydrate conjugates and cinnamic esters), iridoid glycosides (clustered in the groups of terpene glycosides), and iridoid-cinnamic acid glycosides (clustered in the groups of carbohydrate conjugates and terpene glycosides). The chemical makers’ selection of herbal products should not only consider their bioactivity, but also the stability and ease of availability. Therefore, to validate the developed experiment, we focused on the commercially available compounds identified from *S. ningpoensis* extract, including four cinnamic acid glycosides (acteoside, isoacteoside, angoroside C, and castanoside D) found in the high BS cluster (cluster B, **Figure 3**), together with one iridoid glycoside (harpagide) and one iridoid-cinnamic acid glycoside (harpagoside) found in low BS clusters (clusters A and F, **Figure 5**).

**Figure 6:**
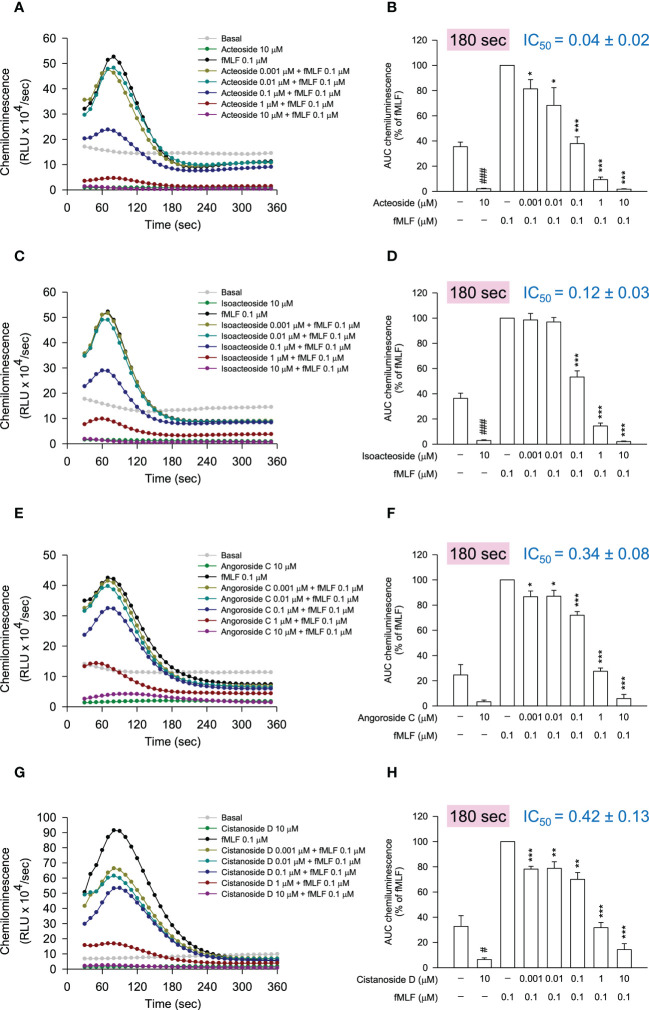
The compounds from the bioactive MIMN clusters inhibited the *N*-formyl methionyl-leucyl-phenylalanine (fMLF)-induced reactive oxygen species (ROS) generation in human neutrophils. **(A, C, E, G)** The luminol-mixed human neutrophils were incubated with DMSO (0.1%, as control) or compounds (0.001–10 μM) and were activated by fMLF (0.1 μM). ROS production was detected by a chemiluminometer. The area under the curve (AUC) value was quantified and shown in **(B, D, F, H)**. The values were shown as the mean ± S.E.M. (n = 3–4). #*p* < 0.05, ###*p* < 0.001 compared with the non-stimulated control; *p < 0.05, **p < 0.01, ***p < 0.001 compared with the stimulated control.

**Figure 7:**
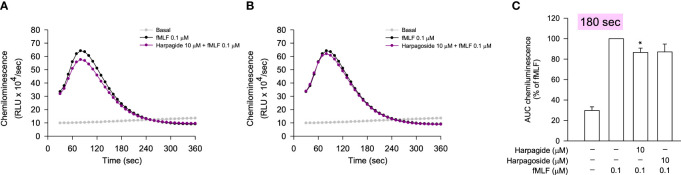
The compounds from the non-bioactive MIMN clusters did not significantly affect the fMLF-induced ROS generation in human neutrophils. **(A, B)** The luminol-mixed human neutrophils were incubated with DMSO (0.1%, as the control) or compounds (0.001–10 μM) and were activated by fMLF (0.1 μM). ROS production was detected by a chemiluminometer. The AUC value was quantified and shown in **(C)**. The values were shown as the mean ± S.E.M. (n = 3–4). *p < 0.05, compared with the stimulated control.

Since some of the selected compounds were found to induce ferricytochrome *c* reduction in the anti-O_2_^•−^ generation assay, the anti-inflammatory evaluation was performed by the detection of reduced reactive oxygen species (ROS) generation in fMLF-stimulated neutrophils using luminol-enhanced chemiluminescence. According to the chemiluminescence response curves (**Figures 6A, C, E, G**) and the area under the curve (AUC) value quantified at 180 seconds (**Figures 6B, D, F, H**), all selected compounds from the bioactive MIMN clusters dose-dependently inhibited the total ROS generation activated by fMLF. The cinnamic acid glycosides with double carbohydrate units (IC_50_: 0.04–0.12 μM) showed more potent anti-inflammatory potential compared with those with triple carbohydrate units (IC_50_: 0.34–0.42 μM). On the contrary, the selected compounds from the non-bioactive MIMN clusters did not show any significant anti-inflammatory effect in activated human neutrophils (**Figure 7**), confirming the analyzing results that cinnamic acid glycosides were the most potent heat-clearing metabolite class.

**Figure 8:**
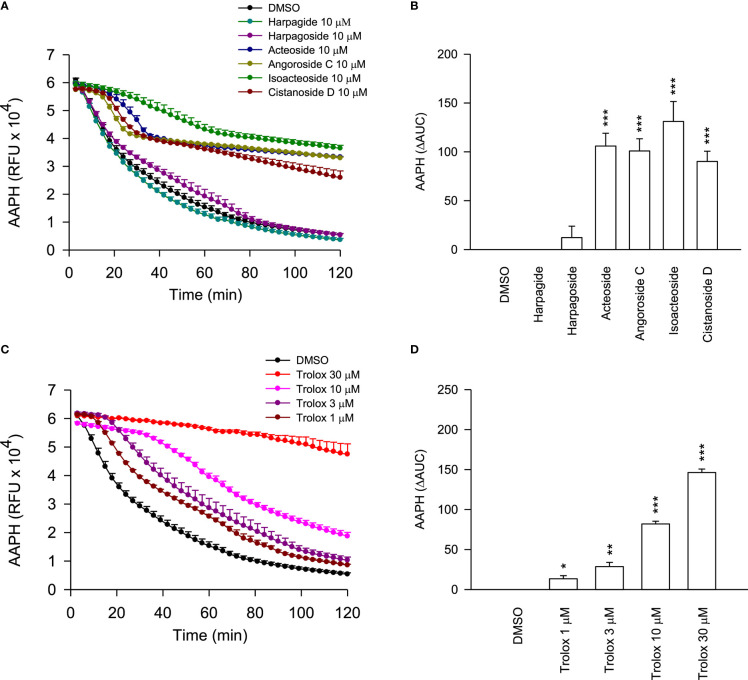
The ROS scavenging properties of *S. ningpoensis* compounds. **(A)** The fluorescence decay curves of 2,20-azobis(2-methylpropionamidine) dihydrochloride (AAPH) in the treatments of DMSO (0.1%, as the control), compounds (10 μM), and **(C)** Trolox (1–30 μM, as the positive control). The AUC value was quantified and shown in **(B, D)**. The values were shown as the mean ± S.E.M. (n = 4). *p < 0.05, **p < 0.01, ***p < 0.001 compared with the control.

To further elucidate the ROS-dependent anti-inflammatory activity of these cinnamic acid glycosides, the oxygen radical absorbance capacity assays were employed. In theFRAP assay (**Table 2**), the cinnamic acid glycosides with double carbohydrate units (acteoside and isoacteoside) exhibited stronger FRAP compared to the triple carbohydrate units (angoroside C and cistanoside D). On the other hand, all cinnamic acid glycosides showed potent ROS scavenging ability in AAPH assay (**Figure 8**). Therefore, we suggested that the anti-inflammatory activities of cinnamic acids from *S. ningpoensis* may be attributed to their oxygen radical scavenging abilities. Harpagide (an iridoid glycoside) and harpagoside (an iridoid-cinnamic acid glycoside) did not show any antioxidant activity in cell-free assays or ROS generation in human neutrophils.

**Table 2:** The effect of *S. ningpoensis* compounds in the ferric reducing antioxidant power (FRAP) assessment.

Compounds	Concentrations	ferricytochrome *c* reduction	
		OD_550_ value	
Harpagide	10 μM	0.006 ± 0.001	
Harpagoside		0.006 ± 0.001	
Acteoside		0.502 ± 0.014	***
Isoacteoside		0.579 ± 0.016	***
Angoroside C		0.009 ± 0.002	*
Cistanoside D		0.015 ± 0.002	**
*α*-Tocopherol	30 μM	0.087 ± 0.009	***

Results are presented as the mean ± S.E.M. (n = 3–6). *p < 0.05, **p < 0.01, ***p < 0.001 compared with the control (DMSO).

**Figure 9:**
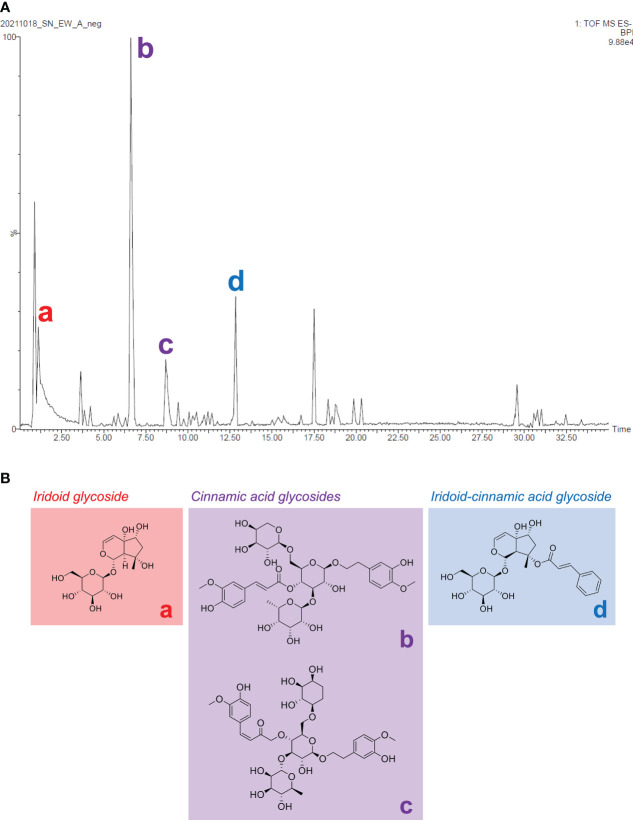
The chemical identification of the major constituents among three major types of compounds from the *S. ningpoensis* extract. **(A)** The MS total ion chromatogram in negative mode; **(B)** the chemical structures of the annotated constituents, harpagide (a), angoroside C (b), isoangoroside C (c), and harpagoside (d).

Harpagide, angoroside C, isoangoroside C, and harpagoside were the four major compounds from the total ion current (TIC) chromatogram of *S. ningpoensis* extract (**Figure 9**). Since cinnamic acid glycosides were analyzed and validated to be the most bioactive chemical class apart from iridoid glycosides and iridoid-cinnamic acid glycosides, the most abundant cinnamic acid glycoside, angoroside C, was suggested as a chemical marker for *S. ningpoensis*.

According to the definition of the Taiwan Herbal Pharmacopeia (Ministry of Health and Welfare, Taiwan, 2021), chemical markers are components used as quantitative indicators to standardize the semi-finished products and finished products of herbal medicines. The selection of chemical markers is mainly based on the biological activity corresponding to the indication of herbal medicine. Therefore, a thorough elucidation of the chemical and biological properties of metabolites present in herbal medicine is needed for chemical markers to be accurately legislated.

In our current study, we schemed a MIMN platform for probing chemical markers of an HCH, *S. ningpoensis*. Three types of glycosides (cinnamic acid glycosides, iridoid glycosides, and iridoid-cinnamic acid glycosides) were illustrated to dominate the metabolic diversity of *S. ningpoensis*. Previous scientific studies revealed several bioactivities of *S. ningpoensis* iridoid glycosides and cinnamic acid glycosides including cardiovascular protective, anti-inflammatory, anti-diabetic, and immunomodulatory activities (Ren et al., 2021). Regarding its bioactivity as a heat-clearance agent, an iridoid glycoside, scropolioside A, was reported to ameliorate the OXA-induced edema in mice. It also inhibited the proliferation of activated T lymphocytes and the production of prostaglandin E₂ (PGE2), leukotriene B4 (LTB4), nitric oxide (NO), interleukin 1β (IL-1β), IL-2, IL-4, tumor necrosis factor α (TNF-α), and interferon γ (IFNγ) *in vitro* (Bas et al., 2007). In murine macrophage raw 264.7 cells, scropolioside A attenuated the inducible nitric oxide synthase (iNOS) and cyclooxygenase-2 (COX-2) expressions and nuclear factor *κ*-light-chain-enhancer of activated B cells (NF-κB). A structure-activity relationship (SAR) study pointed out that the anti-inflammatory activity of *S. ningpoensis* iridoid-cinnamic acid glycosides was highly affected by the substitutions of 6-*O*-substituted cinnamyl moiety (Pasdaran and Hamedi, 2017). Also, the cinnamic acid glycosides including angoroside A, angoroside C, angoroside D, acteoside, and isoacteoside inhibited the release of PGE2 by mouse peritoneal macrophages stimulated with calcium ionophore (DıíAz et al., 2004). Although the anti-inflammatory potential of these glycosides was revealed in various *in vitro* and *in vivo* models, a comprehensive analysis of *S. ningpoensis* anti-inflammatory components from a metabolic perspective is still necessary to answer the question of marker selection.

The developed MIMN-based BS calculation indicated that the cinnamic acid glycosides are the most potent anti-inflammatory chemical class of *S. ningpoensis* as revealed by their effect on ROS inhibition in fMLF-activated neutrophils. Our experimental results also validated and supported the selection of cinnamic acid glycosides as the chemical markers of *S. ningpoensis*.

## 4 Conclusion

The multi-informative molecular networking (MIMN) approach proved to be a promising tool for the future marker selections of herbal medicine. It can provide a more accurate and objective selection of chemical markers by applying novel spectroscopic strategies. Our results indicated that a major cinnamic acid glycoside, angoroside C, was responsible for the heat-clearing effect of *S. ningpoensis* and should be selected as the chemical marker. These results can be implemented for the future amendment of chemical markers in herbal pharmacopeias.

## Data availability statement

The raw data supporting the conclusions of this article will be made available by the authors, without undue reservation.

## Ethics statement

The studies involving human participants were reviewed and approved by The Institutional Review Board (IRB) at Chang Gung Memorial Hospital (protocol code No. 202002493A3). The patients/participants provided their written informed consent to participate in this study.

## Author contributions

L-SW, P-JC, W-CC, Y-CC, K-HL, and T-LH developed the experimental design. K-HL, L-YC, C-HS, B-RP, and Y-CC performed the MS/MS analytical experiments. P-JC and T-LH completed the biological experiments. L-SW, P-TY, K-HL, and T-LH provided reagent and analytical assessment. L-SW, P-JC, W-CC, Y-CC, K-HL, ME-S, and T-LH participated in data interpretation. L-SW, W-CC, K-HL, ME-S, P-JC, and T-LH drafted and revised the manuscript. All authors contributed to the article and approved the submitted version.
