# Supplementary material for: Chemometric-guided chemical marker selection: A case study of the heat-clearing herb Scrophularia ningpoensis
Source: Front Plant Sci. 2023 Mar 17;14:1153710. doi: 10.3389/fpls.2023.1153710 (PMC10088908; doi:10.3389/fpls.2023.1153710)
Supplement: Supplementary file 1 [file DataSheet_1.pdf]

Supplementary Information

Chemometric-Guided Chemical Marker Selection: A Case Study Of The Heat-Clearing Herb *Scrophularia ningpoensis*

Lung-Shuo Wang [1,2], Po-Jen Chen [3,†], Wen-Chi Cheng [4,†], Yu-Chia Chang [5,6,†], Mohamed El-Shazly [7], Lo-Yun Chen [4], Bo-Rong Peng [4], Chun-Han Su [8], Pei-Tzu Yen [9,10], Tsong-Long Hwang [5,6,11,12,13,*] and Kuei-Hung Lai [4,14,15,*]

Contents

Table S1. The Inh% of superoxide generation of the *S. ningpoensis* fractions (20 µg/ml)
Figure S1. The high-performance liquid chromatography (HPLC) bioassay profiling was performed using the retention time-based fractionation approach together with the assessment of neutrophilic inhibition (anti-superoxide anion generation).
Figure S2. The bioactivity score (BS) calculations of selective clusters.

Table S1. The Inh% of superoxide generation of the *S. ningpoensis* fractions (20 μg/ml)

**Sample**	**Superoxide anion** Inh%	
SN-EW-1	6.31±5.23	
SN-EW-2	15.72±6.06	
SN-EW-3	21.19±6.50	*
SN-EW-4	34.94±6.44	**
SN-EW-5	24.36±6.87	*
SN-EW-6	19.01±5.74	*
SN-EW-7	35.38±5.80	**
SN-EW-8	16.80±2.48	**
SN-EW-9	7.08±4.17	

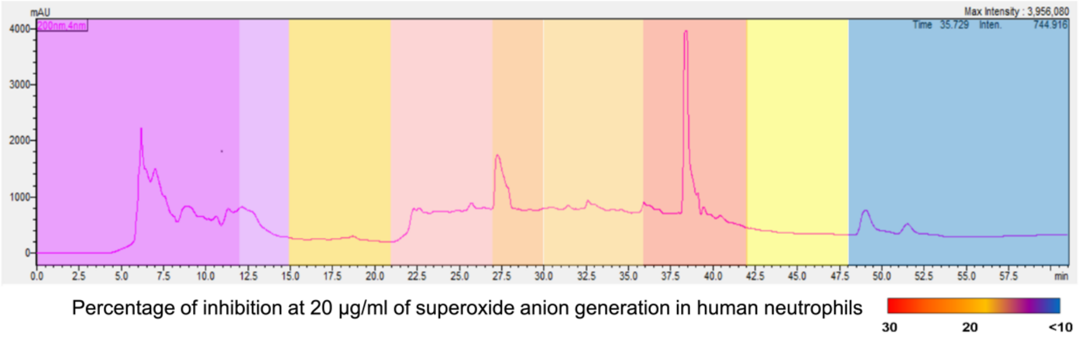

Figure S1. The high-performance liquid chromatography (HPLC) bioassay profiling was performed using the retention time-based fractionation approach together with the assessment of neutrophilic inhibition (anti-superoxide anion generation).

frontiers

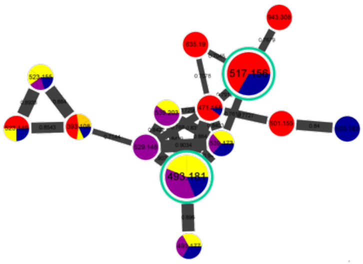

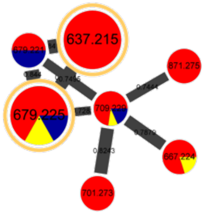

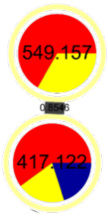

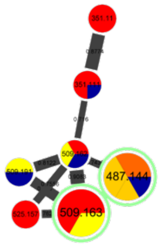

Cluster A (14 nodes)	
Red (>30%)	Pink (>30%)
50	0
45	0
90	0
100	0
67	0
100	0
100	0
BS=552/14=39.43	

Cluster C (7 nodes)	
Red (>30%)	Pink (>30%)
50	0
100	0
67	0
73	0
100	0
100	0
80	0
BS=570/7=81.43	

Cluster G (2 nodes)	
Red (>30%)	Pink (>30%)
67	0
60	0
BS=127/2=63.5	

Cluster D (7 nodes)	
Red (>30%)	Pink (>30%)
100	0
75	0
33	0
100	0
67	0
BS=375/7=53.57	

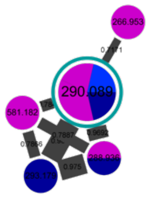

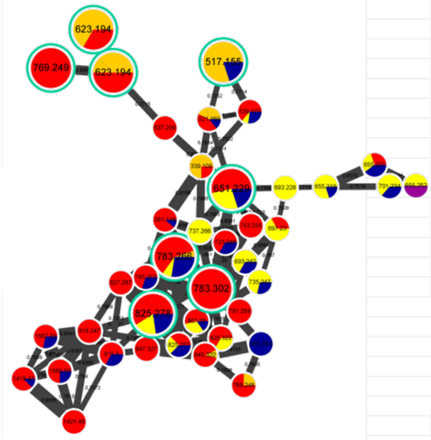

Cluster B (40 nodes)	
Red (>30%)	Pink (>30%)
100	0
33	0
50	0
100	0
43	0
64	0
25	0
53	0
30	0
33	0
100	0
67	0
89	0
60	0
100	0
100	0
100	0
70	0
59	0
54	0
67	0
54	0
75	0
89	0
38	0
100	0
67	0
100	0
100	0
75	0
88	0
71	0
BS=2254/40=56.35	

Cluster E (5 nodes)	
Red (>30%)	Pink (>30%)
0	0
0	0
0	0
0	0
0	0
BS=0/5=0	

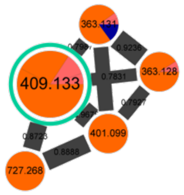

Cluster F (5 nodes)	
Red (>30%)	Pink (>30%)
0	10
0	10
0	16
BS=36/5=7.2	

Figure S2. The bioactivity score (BS) calculations of selective clusters.
